# Targeting glutamine metabolism in hepatic stellate cells alleviates liver fibrosis

**DOI:** 10.1038/s41419-022-05409-0

**Published:** 2022-11-14

**Authors:** Xiaochun Yin, Jin Peng, Lihong Gu, Yan Liu, Xihan Li, Jinhui Wu, Bing Xu, Yuzheng Zhuge, Feng Zhang

**Affiliations:** 1 Department of Gastroenterology, Nanjing Drum Tower Hospital, the Affiliated Hospital of Nanjing University Medical School, Nanjing, Jiangsu China; 2 Hepatobiliary and Pancreatic Center & Liver Transplantation Center, the Affiliated Drum Tower Hospital, Medical School of Nanjing University, Nanjing, China; 3 State Key Laboratory of Pharmaceutical Biotechnology, Chemistry and Biomedicine Innovation Center, Medical School, Nanjing University, Nanjing, 210093 China; 4 Jiangsu Key Laboratory for Nano Technology, Nanjing University, Nanjing, 210093 China

**Keywords:** Liver fibrosis, Energy metabolism

## Abstract

Glutamine metabolism plays an essential role in cell growth, and glutamate dehydrogenase (GDH) is a key enzyme. GDH promotes the metabolism of glutamate and glutamine to generate ATP, which is profoundly increased in multiple human cancers. Through in vitro and in vivo experiments, we verified that the small-molecule GDH inhibitor EGCG slowed the progression of fibrosis by inhibiting GDH enzyme activity and glutamine metabolism. SIRT4 is a mitochondrial enzyme with NAD that promotes ADP ribosylation and downregulates GDH activity. The role of SIRT4 in liver fibrosis and the related mechanisms are unknown. In this study, we measured the expression of SIRT4 and found that it was downregulated in liver fibrosis. Modest overexpression of SIRT4 protected the liver from fibrosis by inhibiting the transformation of glutamate to 2-ketoglutaric acid (α-KG) in the tricarboxylic acid cycle (TCA), thereby reducing the proliferative activity of hepatic stellate cells (HSCs). Collectively, our study reveals that SIRT4 controls GDH enzyme activity and expression, targeting glutamine metabolism in HSCs and alleviating liver fibrosis.

## Introduction

Chronic liver diseases (CLDs) remain life-threatening conditions that also burden society. Liver fibrosis is the major cause of morbidity and mortality in patients with CLD [1]. To date, the mechanisms involved in certain etiologies of liver fibrosis remain to be revealed. Activated hepatic stellate cells (HSCs) play critical roles in various biological processes. For example, liver fibrosis is characterized by the overexpression and deposition of extracellular matrix (ECM)-related proteins produced by activated HSCs and the loss of hepatic parenchymal structure [2]. Strategies to reverse liver fibrosis have been extremely important foci because no effective treatment has been developed to date.

Glutamine is critical for many fundamental functions, such as maintaining mitochondrial metabolism, and is necessary for cell proliferation [3]. Thus, targeting glutamine metabolism is a key potential treatment for liver fibrosis. Du et al. [3] confirmed the importance of glutaminolysis to maintain the activation and proliferation of HSCs and suggested that this process might be a therapeutic target for cirrhosis.

Glutaminase (GLS) is the first enzyme to be activated in glutaminolysis and converts glutamine to glutamate, which can be consumed during protein synthesis and glutathione generation. Early studies showed that blocking glutamine metabolism not only diminished HSCs activation but also decreased the differentiation rate of HSCs [3, 4]. Previous evidence has shown that GLS inhibition may even protect the liver from damage [3]. Glutamate dehydrogenase (GDH) is another key enzyme in glutaminolysis because it converts glutamate to 2-ketoglutaric acid (α-KG), which enters the TCA cycle. GDH expression and enzyme activity can regulate ATP production and cell proliferation [5].

The mammalian sirtuin family comprises seven members, Sirt 1–7, which are involved in regulating various biological functions, such as apoptosis, metabolism, stress responses, aging, differentiation, and cell cycle progression [6]. Mitochondrial protein sirtuin 4 (SIRT4) is a largely uncharacterized member of the sirtuin family and is exclusively found in mitochondria. One key SIRT4 function is related to metabolic regulation. GDH, which converts glutamate to α-ketoglutarate (α-KG) in mitochondria, is regulated by ADP ribosylation mediated by SIRT4 [7]. SIRT4 is highly expressed in important organs, such as the heart, kidney, liver, and brain, under physiological conditions, suggesting that it exhibits important physiological functions. SIRT4 is involved in physiological processes such as energy metabolism [7], insulin secretion [8], and fatty acid oxidation [9]. However, the role of SIRT4 in liver fibrosis and the related mechanisms are unknown.

In this study, we found that glutamine metabolism, especially glutamine catabolism, played an important role in the activation and proliferation of HSCs. Targeting glutamine metabolism with the small-molecule inhibitor EGCG significantly slowed liver fibrosis progression. We conducted a comprehensive analysis of the relationship between SIRT4 expression and glutamine metabolism. Interestingly, we showed that SIRT4 expression was downregulated in liver fibrosis and that SIRT4 exerted antifibrotic effects by regulating glutamine metabolism in HSCs.

## Results

### Glutaminolysis is critical for energy production and anabolism of activated HSCs

**Fig. 1:**
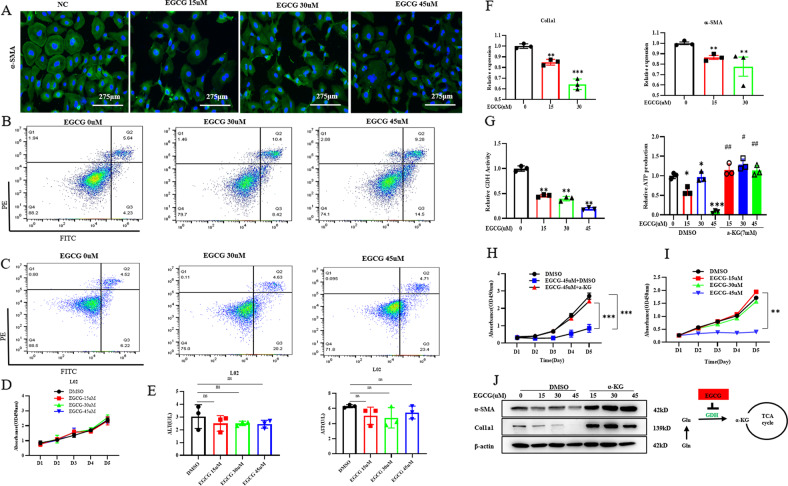
Glutaminolysis is critical for energy production and anabolism of myofibroblastic HSCs. LX-2 cells were grown in complete medium treated with glutaminolysis inhibitors or vehicle (0.1% DMSO) for 1–5 days. **A** Immunofluorescence analysis of α‐SMA expression (scale bars = 275 μm). **B** Effects of EGCG on the apoptosis of LX2 cells. **C** Effects of EGCG on the apoptosis of L02 cells. **D** Cell growth of L02 cells was determined by CCK-8 assay. **E** ALT and AST levels in L02 cells supernatant. **F** Quantitative real‐time PCR analysis of the gene expression of α‐SMA and Col1a1. **G** Glutamate dehydrogenase (GDH) enzyme activity and ATP production. **H**, **I** Cell growth of LX2 cells was determined by CCK-8 assay. **J** Western blot analysis of the protein expression of α‐SMA and Col1a1. The data are reported as the mean ± SD. *P < 0.05, **P < 0.01, and ***P < 0.001 versus the respective control. ^#^*P* < 0.05, ^##^*P* < 0.01, and ^###^*P* < 0.001 versus the respective DMSO groups.

Glutamine metabolism is intrinsically linked to cellular function, and it is taken up by proliferating cells and converted to glutamate. Glutamine is critical to nitrogen metabolism in the liver. The green tea polyphenol epigallocatechin-3-gallate (EGCG) was used to inhibit GDH [10]. After treatment with the GDH inhibitor EGCG, the immunofluorescence staining results showed that EGCG stimulation obviously reduced the expression of α‐SMA (Fig. 1A). The apoptosis of LX-2 cells was significantly increased after EGCG treatment (Fig. 1B). Interestingly, we treated L02 cells with different doses of EGCG for 48 h and measured the effect using cellular apoptosis and CCK-8 assays (Fig. 1C, D). The viability rate of L02 cells was not dose-dependent with EGCG. The cell supernatant was taken to detect the ALT and AST levels (Fig. 1E). Compared with the normal control group, the levels of ALT and AST did not change significantly after treated with different doses of EGCG (*P* > 0.05). These doses of EGCG caused no significant damage to hepatocytes. After treatment with EGCG, LX-2 cells showed decreased expression of Col1α1 and a-SMA (Fig. 1F). We found that GDH enzymatic activity was significantly decreased after treatment with EGCG (Fig. 1G). In addition, the proliferative capacity of the cells was significantly decreased (Fig. 1H) compared to that of vehicle-treated cells. After replenishing the downstream product, a-KG, in the TCA cycle, both the increase in ATP production (Fig. 1G) and the inhibition of LX-2 cell proliferation were reversed (Fig. 1I). After replenishing a-KG, the expression of Col1α1 and a-SMA also increased significantly (Fig. 1J). That is, the proliferative activity of the cells was positively correlated with the ATP energy generated by glutamine metabolism. These data suggested that activated HSCs are highly dependent on glutamine for energy metabolism.

### Glutaminolysis activates myofibroblastic HSCs in acute liver injury

**Fig. 2:**
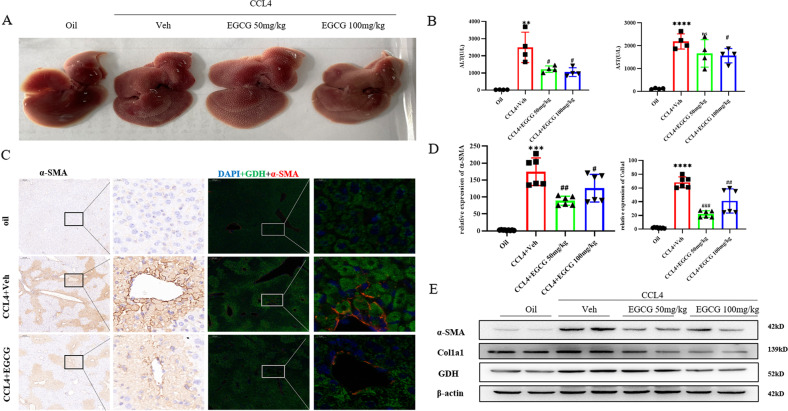
Glutaminolysis activates myofibroblastic HSCs in acute liver injury. **A** Representative images showing the liver morphology in each group at sacrifice. **B** ALT and AST levels in serum. **C** Immunofluorescence and immunohistochemistry analysis of α‐SMA and GDH expression (scale bars = 200 μm or 20 μm). **D** Real‐time quantitative PCR analysis of the mRNA expression of fibrotic genes. **E** Western blot analysis of the protein expression of α‐SMA, Col1a1 and GDH. All the parameters evaluated were significantly decreased after EGCG treatment. The data are reported as the mean ± SD. *p < 0.05; **p < 0.01; ***p < 0.001, ****p < 0.0001 versus the oil group. ^#^*P* < 0.05, ^##^*P* < 0.01, and ^###^*P* < 0.001 versus the CCL4 + Veh group.

We further investigated the antifibrotic effects of EGCG on early fibrogenesis in a CCl4-induced acute liver injury mouse model. For all groups of mice, 6 and 30 h after CCL4 or corn oil intraperitoneal injection, EGCG or normal saline was injected, respectively, and mice were sacrificed 48 h after CCl4 treatment. Levels of biochemical markers of hepatic damage were significantly decreased after treatment with EGCG (50 mg/kg and 100 mg/kg) (Fig. 2A, B). Drug gavage treatment with EGCG induced significant downregulation of early fibrogenesis, as shown by the significant decrease in protein and gene expression of the major extracellular matrix component Col1a1 and HSCs activation markers (α-SMA) in EGCG-treated mice compared to untreated CCl4 mice (Fig. 2D, E). Thus, GDH expression was reduced in the liver tissue of CCL4-induced acute liver injury models, and immunofluorescence staining showed colocalization of GDH and α-SMA in CCL4-induced mouse liver tissue (Fig. 2C). These results suggested that glutaminolysis activated myofibroblastic HSCs in acute liver injury.

### Glutaminolysis activates myofibroblastic HSCs in chronically injured fibrotic livers in mice

**Fig. 3:**
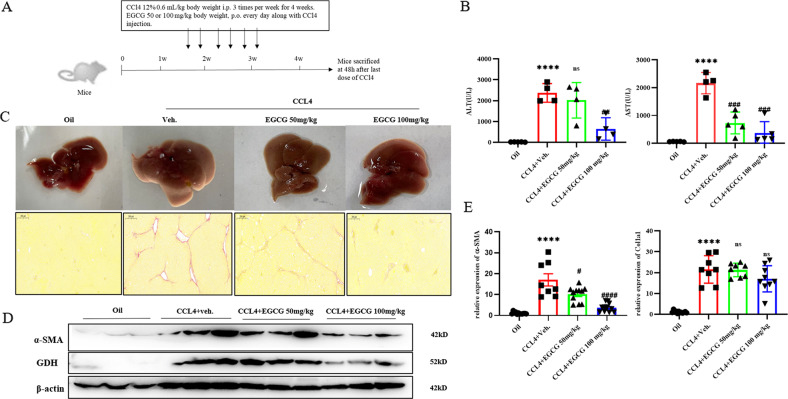
Glutaminolysis activates myofibroblastic HSCs in chronically injured fibrotic livers in mice. **A** Animal experimental procedures. **B** ALT and AST levels in serum. **C** Representative pictures of liver morphology in each group at sacrifice and Sirius red staining (scale bars = 200 μm); red indicates fibrosis. **D** Western blot analysis of the protein expression of α‐SMA, Col1a1, GDH and SIRT4. **E** Real‐time quantitative PCR analysis of the mRNA expression of fibrotic genes. The data are reported as the mean ± SD. *p < 0.05; **p < 0.01; ***p < 0.001; ****p < 0.0001 versus the oil group. ^#^*P* < 0.05, ^##^*P* < 0.01, and ^###^*P* < 0.001 versus the CCL4 + Veh group.

Two doses of EGCG (50 mg/kg and 100 mg/kg) were administered by gavage 3 times per week for 4 weeks, and blood and liver specimens were collected 48 h after the last dose (Fig. 3A). In the EGCG group, liver congestion and necrosis were obviously mitigated at the macroscopic level (Fig. 3A), and serum ALT and AST levels (Fig. 3B) were significantly lower than those in vehicle group mice. Liver specimens were stained with Sirius Red S stain. After CCL4 administration, marked collagen deposition was observed, which was significantly decreased by EGCG treatment (Fig. 3C). In addition to these results, real‐time quantitative PCR and Western blot analysis indicated that fibrotic gene expression decreased significantly after EGCG treatment (Fig. 3D, E), indicating that the progression of fibrosis was significantly slowed. These results showed that blocking glutamine metabolism ameliorated the progression of CCL4-induced fibrosis in mice. Concurrent treatment with EGCG prevented liver fibrosis and collagen fiber deposition in the hepatic parenchyma.

### SIRT4 expression was decreased in liver fibrosis

**Fig. 4:**
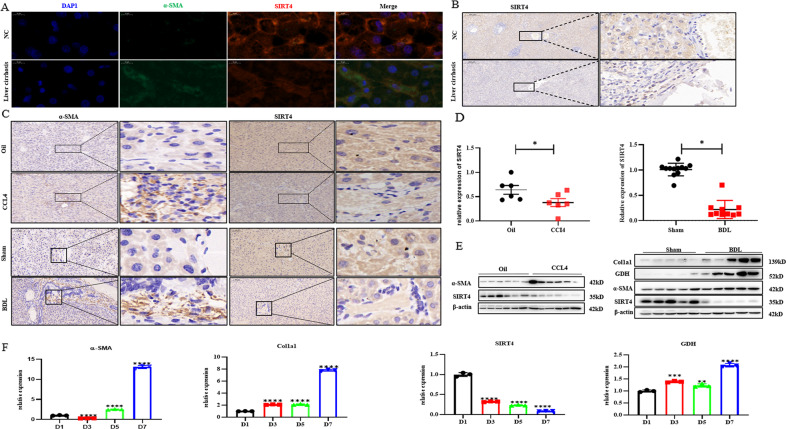
The expression of SIRT4 was decreased in fibrotic livers. **A** Immunofluorescence staining of α-SMA and SIRT4 in liver sections prepared from samples obtained from patients with liver fibrosis or other liver disease (*n* = 6/group; scale bars = 10 μm). **B** Immunohistochemical staining of SIRT4 in liver sections prepared from samples obtained from patients with liver fibrosis or other liver disease (*n* = 6/group; scale bars = 200 μm or 20 μm). **C** Immunohistochemical staining of α-SMA and SIRT4 in fibrotic livers from CCl4‐treated or DBL‐treated mice (scale bars = 100 μm or 10 μm). **D**, **E** SIRT4 expression in fibrotic livers from CCl4‐treated or DBL‐treated mice was detected by real‐time quantitative PCR analysis and Western blot analysis. **F** mRNA levels of α-SMA, Col1a1, GDH and SIRT4 in primary murine HSCs at different days of culture. WT mice were injected with CCl4 or vehicle for 4 weeks. The data are reported as the mean ± SD. *p < 0.05; **p < 0.01; ***p < 0.001; ****p < 0.0001.

We investigated the effects of glutaminolysis on proliferation and phenotype maintenance during HSCs activation. GDH is a downstream effector of SIRT4 [7]. To explore the potential role of SIRT4 in liver fibrosis, we first compared the expression of SIRT4 in different models. Surprisingly, SIRT4 expression was similarly decreased in the liver tissues of patients with fibrosis (Fig. 4A, B). In our study, we demonstrated that the expression of SIRT4 was also markedly decreased in mouse models of liver fibrosis induced by CCl4 injection and BDL (Fig. 4C–E). The SIRT4 expression level was significantly decreased in the BDL and CCL4 treatment groups compared with the control group. GDH expression is higher in liver tissue from mice with liver fibrosis induced by CCl4 injection and BDL. Primary HSCs were isolated from mice and cultured. Primary HSCs were spontaneously activated in vitro, and the expression of a-SMA increased with prolonged culture time. RT‒qPCR analysis showed that GDH was highly expressed in a subset of activated HSCs, but the expression of SIRT4 was decreased (Fig. 4F). We are the first to discover that the expression of SIRT4 in activated HSCs in the liver is reduced and that overexpression of SIRT4 can significantly inhibit the proliferation of HSCs and reverse liver fibrosis.

### Overexpression of SIRT4 inhibited the proliferation of HSCs and decreased ECM deposition

**Fig. 5:**
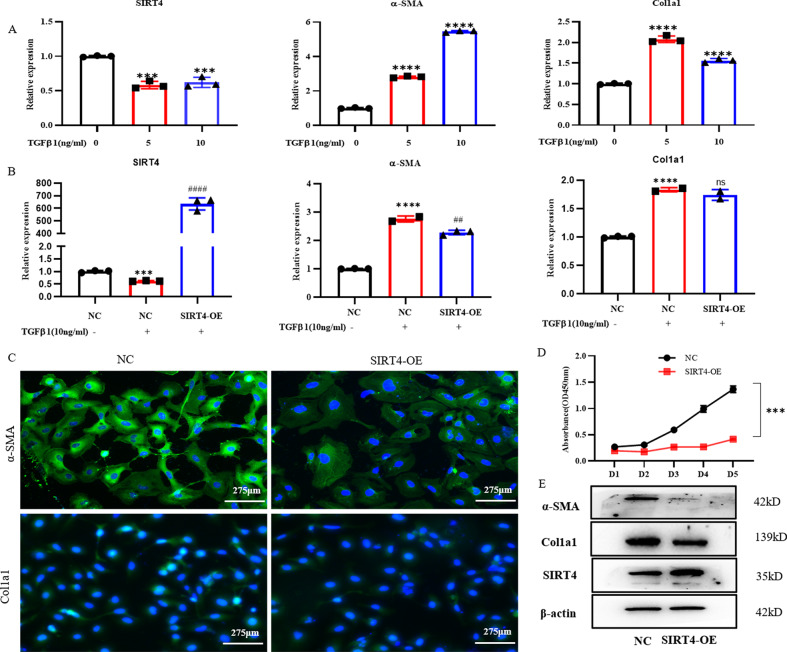
Overexpression of SIRT4 ameliorated HSCs activation. **A**, **B** Real‐time quantitative PCR analysis of the mRNA expression of fibrotic genes. **C** Immunofluorescence analysis of α‐SMA expression (scale bars = 275 μm). **D** Cell counting kit-8 (CCK-8) analysis of cell viability. **E** Western blot analysis of the protein expression of α‐SMA, Col1a1, and SIRT4. The data are reported as the mean ± SD. *p < 0.05; **p < 0.01; ***p < 0.001; ****p < 0.0001 versus the respective control. ^#^*P* < 0.05, ^##^*P* < 0.01, ^###^*P* < 0.001, and ^####^*P* < 0.0001 versus the NC + TGFβ1 group.

The loss of SIRT4 expression in liver fibrosis prompted us to determine whether overexpression of SIRT4 can inhibit the activation of HSCs. To validate the underlying mechanisms of SIRT4 action in liver fibrosis, we treated LX-2 cells with TGF-β1 to activate them in vitro. As expected, TGF-β1 considerably decreased the cellular RNA levels of SIRT4 (Fig. 5A). The cellular mRNA levels of both collagen type 1 and α-SMA were drastically increased by TGF-β1 (Fig. 5A), whereas pretransfection with a SIRT4 overexpression plasmid significantly reduced the protein level of α-SMA (Fig. 5E). To investigate the role of SIRT4 in liver fibrosis, we overexpressed SIRT4 in LX2 cells. In culture‐activated LX-2 cells, SIRT4 overexpression greatly reduced the mRNA expression of α‐SMA (Fig. 5B). Hence, the immunostaining results supported the inhibitory effects of SIRT4 on liver fibrosis (Fig. 5C). Overexpression of SIRT4 also inhibited the proliferation and viability of LX‐2 cells (Fig. 5D). Western blot analysis validated the finding showing that SIRT4 reduced α-SMA and Col1a1 expression in LX-2 cells compared with the control group (Fig. 5E)

### SIRT4 alleviates liver fibrosis by regulating glutamine metabolism

**Fig. 6:**
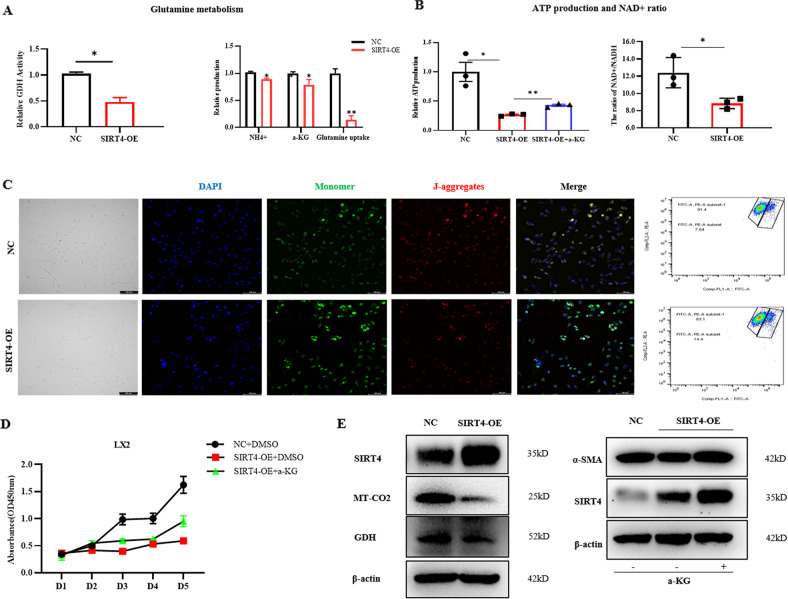
SIRT4 alleviates HSCs activation by regulating glutamine metabolism. GDH activity and secreted a-KG and NH4+ levels were measured 48 h after SIRT4 overexpression plasmid transfection in LX-2 cells. **B** The ATP and NAD+ levels decreased after SIRT4 overexpression. **C** The mitochondrial membrane potential decreased after SIRT4 overexpression, as shown by the JC-1 kit assay (scale bars = 100 μm). Blue represents nuclei, green represents monomers, and red represents polymers. **D** Cell viability was determined by CCK-8 assay. **E** GDH and the mitochondrial marker MT-CO2 levels were significantly reduced after SIRT4 overexpression, as shown by Western blot analysis. The data are representative of the mean ± SD. *P < 0.05; **P < 0.01; ***P < 0.001; ****P < 0.0001 versus the respective control.

**Fig. 7:**
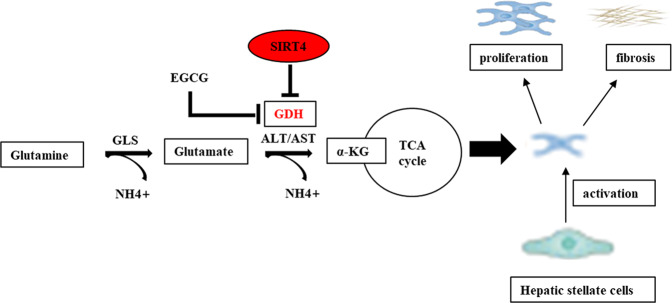
Graphical abstract. SIRT4 controls GDH enzyme activity, targeting glutamine metabolism in HSCs, inhibiting the activation and proliferation of HSCs, and ultimately alleviating liver fibrosis. Glutamine metabolic pathway: Glutamine is catalyzed by mitochondrial glutaminase (GLS) to produce glutamate and NH4+; Glutamate is converted into α-Ketoglutaric acid (α-KG) under the catalysis of GDH and enters the tricarboxylic acid (TCA) cycle, providing energy for the rapidproliferation of cells.

We further explored the downstream mechanisms underlying the effects of SIRT4 on liver fibrosis. In this study, we performed plasmid construction-induced overexpression of SIRT4 in LX-2 cells and confirmed the transfection effect by RT‒qPCR analysis. We found that SIRT4 inhibits glutamine metabolism by reducing GDH enzyme activity. Moreover, SIRT4-overexpressing cells exhibited significantly decreased glutamine uptake and α-KG and NH4^+^ production (Fig. 6A), indicating that SIRT4 decreased the ability of these cells to utilize glutamine for mitochondrial energy production, as described in other studies [7, 11, 12]. Similar outcomes were detected in our experiments with SIRT4-overexpressing LX-2 cells (Fig. 6B). Additionally, overexpression of SIRT4 suppressed GDH gene expression in myofibroblastic HSCs. Hence, we proposed that SIRT4 alleviates liver fibrosis by regulating glutamine metabolism reprogramming. Mitochondrial function was assessed by JC-1 staining (Fig. 6C). JC-1 accumulated in the mitochondrial matrix to form polymers when the mitochondrial membrane potential was relatively high and emitted red fluorescence under a fluorescence microscope. Conversely, JC-1 was monomeric and emitted green fluorescence under a fluorescence microscope. The green fluorescence in cells from the SIRT4-overexpressing group was significantly higher than that in cells from the control group, and the mitochondrial membrane potential decreased, suggesting impaired mitochondrial function and decreased ATP production (Fig. 6B, C). Functional overexpression of SIRT4 suppressed cell proliferation that was rescued by a-KG supplementation (Fig. 6D). Correspondingly, the expression of α-SMA decreased by SIRT4 overexpression could be attenuated after α-KG replenishment. In addition, western blot experiments showed that overexpression of SIRT4 reduced the levels of GDH and mitochondria-related proteins (Fig. 6E). In conclusion, these findings suggested that SIRT4 alleviates liver fibrosis by regulating glutamine metabolism (Fig. 7).

## Discussion

In this study, we described the protective role played by SIRT4 in the pathogenesis of liver fibrosis. SIRT4 controls GDH enzyme activity and expression, regulating glutamine metabolism to inhibit HSCs proliferation. Therefore, regulating SIRT4 expression may be effective in treating patients with fibrosis.

Metabolic reprogramming activates glutaminolysis to fuel the TCA cycle in mitochondria of rapidly proliferating cancer and myofibroblastic cells [3, 13]. SIRT4 regulates glutaminolysis in mitochondria by repressing GDH activity [7, 11]. However, the role of SIRT4 in liver fibrosis is not clear. To explore the precise function of SIRT4 in the pathogenesis of liver fibrosis, we generated SIRT4-overexpressing human HSCs. We found that overexpression of SIRT4 significantly decreased the expression of fibrotic markers such as α-SMA and the proliferation level of activated LX-2 cells. In contrast, the expression of SIRT4 was significantly reduced compared with that in normal liver tissue in both murine liver fibrosis models and human liver samples. Together, these results implicated a protective role for SIRT4 against fibrosis. To date, SIRT4 has mostly been studied in metabolic diseases and cancer [14–17]. In tumor-related studies, it has been proposed that SIRT4 is strongly correlated with glutamine metabolism, playing an important role in maintaining energy metabolism and contributing to the energy required for rapid tumor cell proliferation [11, 14]. Recent studies have shown that SIRT4 is involved in a wide range of mitochondrial metabolic processes and plays an important role in metabolism, energy homeostasis, stress response and longevity [9, 11, 18]. Another study found that SIRT4 upregulation exhibited the potential to counteract HFD-induced lipid accumulation, inflammation, and fibrogenesis [19]. However, the role of SIRT4 in liver fibrosis remains unclear. However, our study advances our understanding of the function of SIRT4 in liver fibrosis, especially in HSCs.

In this study, we clarified the expression of SIRT4 in vivo. One novel aspect of our study indicated that the expression of SIRT4 was lower in the liver of mice with fibrosis, which led to inhibited acquisition of the fibrotic phenotype by HSCs in vitro, which was consistent with previous research results [20]. In addition, we confirmed an important role for glutamine metabolism in HSCs proliferation and phenotype maintenance. In this study, we show that HSCs transdifferentiation was characterized by the simultaneous induction of glutaminolysis to meet the high energy demands associated with cell proliferation and ECM production. Several studies have shown that transdifferentiation of HSCs into the activated form involves reprogramming of energy metabolism, including glycolysis and glutaminolysis [3, 13]. Glycolytic and mitochondrial metabolism are increased in activated HSCs [13, 21, 22]. The expression and activity of key enzymes related to energy metabolism are also increased significantly [23]. Thus, metabolic shifts associated with HSCs transdifferentiation revealed novel and potent targets for the treatment of liver fibrosis. Although SIRT4 functions as an ADP-ribosyltransferase and deacetylase [7, 17], we speculate that SIRT4 may inhibit glutaminolysis in activated HSCs during liver fibrosis through a GDH-dependent pathway.

The role played by glutamine metabolism, in which GDH is particularly important, has not been extensively characterized. Reprogramming of energy metabolism is important to liver disease [13, 24]. Our study deeply explored the process of liver fibrosis to determine whether it was significantly slowed after GDH inhibition both in vitro and in vivo. The important role played by glutamate in HSCs in various liver diseases has been recently reported. Choi et al. [25] found that the uptake of glutamate was increased in activated HSCs and that mGluR5 activation enhanced the cytotoxicity of NK cells. GDH is a key enzyme in glutamine metabolism. Glutamate is converted into α-KG under the catalysis of GDH and enters the TCA cycle in mitochondria, providing energy for the rapid proliferation of cells [26, 27]. In several current studies, the glutamine metabolism inhibitor EGCG has been used to inhibit GDH activity [10, 11, 28]. EGCG, a major constituent of green tea, may protect against NAFLD initiation and development by alleviating oxidative stress and the related metabolism [29]. A potential adverse effect of high-dose EGCG is hepatotoxicity [30]. In our study, the viability rate of L02 cells was not dose-dependent with EGCG, and these doses of EGCG did not cause obvious damage to hepatocytes. However, the same dose of EGCG can inhibit the proliferation of activated HSCs. In the present study, EGCG significantly inhibited the expression of GDH and slowed the progression of liver fibrosis. The small-molecule EGCG, a GDH enzyme inhibitor, effectively blocked the effect of glutamine metabolism on HSCs and reversed the inhibitory cell proliferation effect after replenishing α-KG.

In particular, three sirtuins, SIRT3, SIRT4, and SIRT5, are located within the mitochondrial matrix, where they regulate energy production and antioxidant pathways [6, 31]. As a mitochondrial sirtuin, SIRT4 is associated with mitochondrial function and ATP production [32, 33]. Similarly, we found that after the upregulation of SIRT4, the mitochondrial membrane potential of activated LX-2 cells was reduced, and mitochondrial function was impaired. Correspondingly, mitochondrial ATP production was reduced, and the NAD^+^/NADP ratio was reduced.

Our findings thus highlight the unique effect of SIRT4 on suppressing the progression of fibrosis by modulating glutamine metabolism. The present study also indicated that SIRT4 plays an inhibitory role during liver fibrosis by regulating glutamine energy metabolism. GDH promotes the metabolism of glutamate and glutamine, generating ATP, and this process can be regulated by SIRT4. Upregulation of SIRT4 gene expression effectively blocked the effect of glutamine metabolism on LX-2 cells and reversed the inhibitory cell proliferation effect induced by SIRT4 after replenishing α-KG, suggesting that SIRT4 acts mainly on HSCs by regulating glutamine energy metabolism. Previous studies [7, 11, 12, 18] confirmed that SIRT4 targets GDH to induce ADP-dependent ribosyltransferase activity, thereby inhibiting glutamine metabolism, limiting ATP production, and inhibiting cell growth. Our study describes, for the first time, the regulatory effect of SIRT4 on GDH enzyme activity in HSCs and demonstrates that SIRT4 inhibits glutamine metabolism in HSCs in a mechanism similar to that of tumor metabolic reprogramming and plays an antifibrotic role. In future research on liver fibrosis-related diseases, investigations into therapeutics should include analyses of glutamate metabolism and SIRT4. Sirtuins are enzymes that can be manipulated by small molecules. Therefore, developing therapeutics against fibrosis through SIRT4-related pathways in the cell is an interesting research direction.

In conclusion, in the current work we demonstrated that modest overexpression of SIRT4 in the liver protected against fibrosis by inhibiting the transformation of glutamate into α-KG in the TCA cycle, thereby reducing the proliferative activity of HSCs and alleviating the development of liver fibrosis (Fig. 7). These findings may provide novel ideas for the management of liver fibrosis. We also expect that more studies will be designed to validate our findings and provide more evidence on the molecular mechanisms involved in the pathogenesis of liver fibrosis.

## Methods

### Human liver samples

Human liver samples were collected from patients with fibrosis or other liver diseases (peripheral tissues in liver hemangioma) in the Nanjing Drum Tower Hospital, the Affiliated Hospital of Nanjing University Medical School. This study was performed in accordance with the principles outlined in the Declaration of Helsinki and approved by the Nanjing Drum Hospital Clinical Research Ethics Committee. Informed consent for tissue analysis was obtained before liver biopsy or surgery.

### Animal experiments

Eight-week-old male BALB/c mice (weighing 20–22 g) were purchased from Nanjing Junke Bioengineering Corporation, Ltd. (NanJing City, JiangSu Province, China). The livers were rapidly excised and weighed. All animal studies were performed according to the guidelines of the Institutional Animal Use and the Animal Experimentation Ethics Committee of Nanjing Drum Tower Hospital, the Affiliated Hospital of Nanjing University Medical School. Liver fibrosis was induced by injecting mice with 0.6 mL/kg carbon tetrachloride (CCl4) 3 times per week for 4 weeks. For the BDL (bile duct ligation) mouse models, the mice were subjected to sham surgery or high bile duct ligation surgery under anesthesia. No blinding was applied in either in vivo experiment. To identify the pharmacological effect of GDH inhibition, mice were treated with EGCG (#HY-13653, MCE; 50 mg/kg body weight, p.o., every day) along with CCl4 injection. The animals were randomly divided into four groups (*n* = 6–8 in each group): (1) control with the administration of vehicles only (Corn oil); (2) CCl4 (12%, 0.6 ml/kg in Corn oil) + vehicles (normal saline); and (3) CCl4 (12%, 0.6 ml/kg in Corn oil) + EGCG (50 mg/kg in normal saline). (4) CCl4 (12% 0.6 ml/kg in corn oil) + EGCG (100 mg/kg in normal saline). Mice were sacrificed 48 h after the last dose was administered, and liver tissues were fixed with 4% paraformaldehyde.

### Isolation and culture of primary HSCs

Briefly, the mice were anesthetized by pentobarbital sodium injections and perfused via the portal vein with successive pronase E (0.4 mg/ml, Sigma, USA) and collagenase-IV (0.5 mg/ml, Roche, Germany) solutions. The liver tissue was isolated and digested with collagenase IV (1 mg/ml) and DNase (0.02 mg/ml, Roche, Germany) in vitro. The tissue was then filtered through a 100 μm mesh. Cells were separated using Nycodenz gradient (Accurate Chemical, USA) centrifugation. Primary HSCs were seeded in DMEM with 10% FBS, 100 U/ml penicillin sodium, and 100 μg/ml streptomycin sulfate and incubated at 37 °C in CO2.

### Cell line treatment

LX-2 [34], an immortalized human HSCs cell line (Procell, Wuhan, China), was cultured in glutamine-containing RPMI 1640 (Bio-Channel) supplemented with 10% fetal bovine serum. The human liver cell line L02 (Procell, Wuhan, China) was cultured in Dulbecco’s modified Eagle’s medium (DMEM) with 10% fetal bovine serum (Bio-Channel). The cells were incubated at 37 °C in a 5% humidified CO_2_ atmosphere. Overexpression of SIRT4 was achieved using plasmids (#G0180974-2, Ibsbio), and transfection of SIRT4 plasmid/empty vector controls was performed using Lipofectamine 2000 (Thermo Fisher Scientific) according to the manufacturer’s instructions for 48 h in 6-well plates. After 72 h, the cells were treated with G418 (Selleck., MO, USA) to select stably transfected clones. The transfected cells were treated with EGCG (MCE, HY-13653) or 2-KG (CAS#328-50-7) for 48 h or treated with TGFβ1 (MCE, HY-P7118) for 24 h.

### Western blot analysis

Protein isolation and Western blotting were carried out as described previously [35]. The following primary antibodies were used: anti-SIRT4 (1:1,000, Invitrogen, # PA5-114377), anti-α‐SMA, anti-collagen I and anti-‐actin rabbit monoclonal antibodies (mAbs, 1:1,000; Proteintech). The secondary antibodies, including HRP‐conjugated goat anti‐rabbit immunoglobulin G (IgG) or goat anti‐mouse IgG (Cell Signaling Technology, MA, United States), were diluted 1:2000.

### Quantitative real-time PCR

Total RNA was extracted from the livers and HSCs using TRIzol reagent (TaKaRa, Kusatsu, Japan) according to the manufacturer’s specifications. Reverse transcription-PCR and real-time quantitative PCR analyses were performed as described by Zhang et al. [35]. The primer sequences used are shown in the Supplementary Material: Supplementary Table 1.

### Cell proliferation experiments

A total of 4000 cells/well were plated 12 h before treatment in 96-well plates. Following overnight culture at 37 °C, transfection was performed. Cell viability was evaluated after 1–5 days of transfection with Cell Counting Kit-8 (Dojindo, Kumamoto, Japan) according to the manufacturer’s instructions.

### Immunofluorescence

Cells treated with different reagents were seeded in 24-well plates at a density of 1 × 10^3^ cells per well. Seventy-two hours after seeding, the cells were fixed with 4% paraformaldehyde for 15 min at room temperature and permeabilized with 0.2% Triton X-100 for 15 min. After blocking with 5% bovine serum albumin (BSA) for 1 h at room temperature, the cells were incubated with antibodies against α-SMA (1:100, Proteintech, #14395-1-AP) and collagen-I (1:200, Proteintech, # 14695-1-AP) overnight at 4 °C. The cells were washed 3 times and then incubated with Alexa Fluor® 488 (Abcam, ab150077) or Alexa Fluor® 647 (Abcam, 150115) for 1 h at room temperature in the dark. Then, the cells were incubated with DAPI (Beyotime, C1005) for 20 min. The cells were visualized with a fluorescence microscope (Olympus, Tokyo, Japan).

### JC-1 analysis for mitochondrial membrane potential

Mitochondrial membrane depolarization was monitored by changes in the tetraethyl-benzimidazolyl carbocyanine iodide (JC-1) (Beyotime, C2006) green: red fluorescence ratio, where an increased ratio is indicative of an increase in mitochondrial membrane potential (MMP). The increased ratio may be a landmark of the early stage of apoptosis. Cells were incubated with JC-1 (1:1,000 dilution) for 20 min at 37 °C. Then, the cells were harvested, washed twice with 1× washing buffer and mixed in 100 μL of 1× washing buffer. The fluorescence intensity was measured by flow cytometry.

### ATP assay

Cells treated with different reagents were collected into 1.5-mL tubes and pelleted by centrifugation. After the cells were washed twice with PBS buffer, 200 μL of lysis buffer from an ATP detection kit was added to each tube, and then, the cells were lysed according to the manufacturer’s instructions (Beyotime, S0026). The lysate was centrifuged at 12,000 × *g* for 5 min at 4 °C. The supernatants were poured into new 1.5-mL tubes for ATP testing with an ATP detection kit.

### Cell apoptosis

Cellular apoptosis was quantified by flow cytometry assay after annexin V–FITC and propidium iodide (PI) staining. According to the instructions, the cells were detached with 2.5% trypsin-EDTA and centrifuged at 1500 rpm for 5 min. The pellet was suspended in 300 µL of 1X binding buffer (FITC Annexin V Apoptosis Detection Kit I, BD Biosciences, USA). Flow cytometry was performed on a BD FACSCalibur flow cytometer, and data analysis was performed with the accompanying software.

### Enzymatic activity assay

GDH activity was measured with a glutamate dehydrogenase activity colorimetric assay kit (BioVision, Milpitas, Calif., USA) according to the manufacturer’s protocol. Briefly, transfected cells (1 × 10^6^) were homogenized. Glutamate was added to the cells, and the NADH produced by GDH was measured with a Model 680XR Microplate Reader at 450 nm.

### Measurement of NADP+/NADPH ratios

The intracellular levels of NADP+ and NADPH were measured with an NADP+/NADPH assay kit (Beyotime, Cat# S0179). Briefly, samples isolated from 1 × 10^6^ cells with 200 μL of NADP^+^/NADPH extraction buffer were heated at 60 °C for 30 min to decompose NADP^+^, and then, G6PDH working solution was added dropwise to convert NADP+ to NADPH, and NADPH developer was added. Finally, the absorbance was read at OD450 nm.

### Statistical analysis

All images are representative of at least 3 independent experiments. GraphPad Prism 8 (San Diego, USA) was used to perform statistical analysis. One-way analysis of variance (ANOVA) or a T test were performed. A *p* value < 0.05 was accepted as significant and is shown by a single asterisk (*) when *p* < 0.05, two asterisks (**) when *p* < 0.01 and three asterisks (***) when *p* < 0.001. The standard deviation (SD) was shown as error bars.

## Supplementary information

Original Data File

Primer used for qRT-PCR

Checklist

## Data Availability

The datasets generated during and/or analysed during the current study are available from the corresponding author on reasonable request.
