# Supplementary material for: Targeting glutamine metabolism in hepatic stellate cells alleviates liver fibrosis
Source: Cell Death Dis. 2022 Nov 14;13(11):955. doi: 10.1038/s41419-022-05409-0 (PMC9663710; doi:10.1038/s41419-022-05409-0)
Supplement: Supplementary file 1 — Original Data File [file 41419_2022_5409_MOESM1_ESM.docx]

**Fig.1C**

**β-actin α-SMA Col1a1**









**Fig.2E**

**β-actin α-SMA** **Col1a1 GDH**











**Fig.3D**

**β-actin α-SMA GDH**









**Fig.4D**

**β-actin SIRT4 α-SMA**









**β-actin SIRT4 α-SMA Col1a1**











**GDH**





**Fig.5E**

**β-actin SIRT4 Col1a1** **α-SMA**











**Fig.6E**

**β-actin SIRT4 MT-CO2 GDH**











**Fig.6F**

**β-actin SIRT4 α-SMA**
