# Supplementary material for: Targeting glutamine metabolism in hepatic stellate cells alleviates liver fibrosis
Source: Cell Death Dis. 2022 Nov 14;13(11):955. doi: 10.1038/s41419-022-05409-0 (PMC9663710; doi:10.1038/s41419-022-05409-0)
Supplement: Supplementary file 2 — Primer used for qRT-PCR [file 41419_2022_5409_MOESM2_ESM.docx]

**Supplementary Table 1. Primer used for qRT-PCR**

|  | **Gene symbol** | **Primer Forward** | **Primer Reverse** |
| --- | --- | --- | --- |
| **Human** | *β-actin* | GGCACCACACCTTCTACAATGAG | GGATAGCACAGCCTGGATAGCA |
|  | *α-SMA* | CCTGTGTTGTGGTTTACACTGG | GGGGGAATTATCTTTCCTGGTCC |
|  | *Col1a1* | GAGGGCCAAGACGAAGACATC | CAGATCACGTCATCGCACAAC |
|  | *SIRT4* | GCTTTGCGTTGACTTTCAGGT | CCAATGGAGGCTTTCGAGCA |
|  | *GDH* | CACTCTGGCTTGGCATACAC | CTCAGGTCCAATCCCAGGTT |

| **Mouse** | *β-actin* | AGCCATGTACGTAGCCATCC | CTCTCAGCTGTGGTGGTGAA |
| --- | --- | --- | --- |
|  | *α-SMA* | TCCCTGGAGAAGAGCTACGAACT | AAGCGTTCGTTTCCAATGGT |
|  | *Col1a1* | GAGCGGAGAGTACTGGATCG | GTTCGGGCTGATGTACCAGT |
|  | *SIRT4* | TTTCCTCTGAGTTCCGCTGCTCAA | AAGGCGACACAGCTACTCCATCAA |
